# ﻿A missing piece is found: a new species of *Paepalanthus* (Poales, Eriocaulaceae) and the puzzling relations of the campos rupestres mountaintop floras of eastern Minas Gerais, Brazil

**DOI:** 10.3897/phytokeys.242.122824

**Published:** 2024-06-11

**Authors:** Luiz Henrique Rocha, Paulo Minatel Gonella, Caroline Oliveira Andrino

**Affiliations:** 1 Universidade Federal de São João del-Rei (UFSJ), Departamento de Ciências Exatas e Biológicas, CEP 35701-970, Sete Lagoas, Minas Gerais, Brazil Universidade Federal de São João del-Rei Sete Lagoas Brazil; 2 Universidade Estadual Paulista (UNESP), Faculdade de Ciências, Departamento de Ciências Biológicas, CEP 17033-360, Bauru, São Paulo, Brazil Universidade Estadual Paulista São Paulo Brazil; 3 Universidade de Brasília (UnB), campus Darcy Ribeiro, Departamento de Botânica, CEP 70910-900, Brasília, Distrito Federal, Brazil Universidade de Brasília Brasília Brazil

**Keywords:** Atlantic Forest, endemism, João Pinto Formation, Serra do Padre Ângelo, threatened species, Espécie ameaçada, endemismo, Formação João Pinto, Mata Atlântica, Serra do Padre Ângelo

## Abstract

*Paepalanthus* is a diverse genus characteristic of the campos rupestres, a megadiverse vegetation found on mountaintops of mainly quartzitic mountain ranges of central-eastern Brazil. Recent efforts on prospecting the biodiversity of Serra do Padre Ângelo, a small mountain complex in eastern Minas Gerais, yielded several new plant and animal species, highlighting the urgency of conservation actions towards this still unprotected area. Here, we describe yet another new species found in the campos rupestres of these mountains, *Paepalanthusmagnus*, a mountaintop microendemic species morphologically similar to taxa found in the Espinhaço Range, over 200 km distant, a biogeographic pattern shared by several other species. The affinities of the new species are discussed, and we provide illustrations, photographs, and SEM photomicrographs of the seed. We also discuss the conservation status of the species, which is preliminarily assessed as Critically Endangered, reinforcing the urgent need to address the conservation of the unique biodiversity of Serra do Padre Ângelo.

## Abstract (es)

*Paepalanthus* é um gênero característico dos campos rupestres, uma vegetação megadiversa encontrada no topo de montanhas de cadeias montanhosas, principalmente quartzíticas, do centro-leste do Brasil. Esforços recentes de inventariar a biodiversidade da Serra do Padre Ângelo, um pequeno complexo montanhoso no leste de Minas Gerais, resultaram na descoberta de várias novas espécies de plantas e animais, destacando a urgência de ações de conservação para esta área ainda não protegida. Aqui, descrevemos mais uma nova espécie encontrada nos campos rupestres dessas montanhas, *Paepalanthusmagnus*, uma espécie microendêmica, morfologicamente semelhante a táxons encontrados na Serra do Espinhaço, a mais de 200 km de distância, um padrão biogeográfico repetido por várias outras espécies. As afinidades da nova espécie são discutidas e fornecemos ilustrações, fotografias da planta e de microscopia eletrônica de varredura (MEV) da semente. Também discutimos o status de conservação da espécie, que é preliminarmente avaliada como Criticamente Em Perigo, reforçando a necessidade urgente de abordar a conservação da biodiversidade única da Serra do Padre Ângelo.

## ﻿Introduction

Eriocaulaceae Martinov is a monocotyledon family belonging to the Poales (APG IV 2016), which comprises around 1400 species divided into 18 genera (Giulietti et al. 2012; Andrino et al. 2023; Sano et al. 2024) and two subfamilies: Eriocauloideae Ruhland and Paepalanthoideae Ruhland (Ruhland 1903). It has a pantropical distribution, but an important center of diversity is found in the campos rupestres of east Brazil, especially in the Espinhaço Range (ER), where the family represents around 10% of the diversity of vascular plants, with an endemism rate of 85% (Costa et al. 2008, 2018). *Paepalanthus* Mart. is the most representative genus of the family in the campos rupestres (Giulietti et al 1997), however, its traditional circumscription, which once included more than 400 species (Ruhland 1903), no longer adequately represents the diversity of the genus. It was found to be polyphyletic and lacked diagnostic characters, prompting a recent proposal for a new classification by Andrino et al. (2023). In this new classification, *Paepalanthus* is delimited as a monophyletic genus comprising 256 species, and the remaining species were segregated into eight new genera, in addition to *Actinocephalus* (Körn.) Sano, *Tonina* Aubl., and *Lachnocaulon* Kunth, previously nested within *Paepalanthus* (Andrino et al. 2023). According to the new circumscription, *Paepalanthus* is characterized by having trimerous and isostemonous flowers, the pistillate flowers with free petals and free stigmatic branches, and the seed coat presenting rectangular or narrow hexagonal cells with appendices with truncate apex or with a “T” form along the periclinal walls, and lacking micropapillae (Andrino et al. 2023).

Although re-circumscribed, *Paepalanthus* remains one of the most speciose genera of Eriocaulaceae, with an elevated rate of microendemic species exclusive to a single mountaintop, with around 82% of the ca. 200 species that occur in the Espinhaço Range being endemic to its campos rupestres (Costa et al. 2008). Such endemism is a combination of various factors, including the fragmented landscapes of the Espinhaço Range (ER) and other quartzitic massifs, isolating plant populations among areas of complex topography characterized by dystrophic soils. Moreover, past climate fluctuations and the reduced dispersal capacity of species have also played crucial roles in this process contributing to speciation, resulting in the emergence of new species that are isolated on mountaintops (Vasconcelos et al. 2020).

Recently, species of Eriocaulaceae were identified in small quartzitic mountains in the Doce River basin, located around 200 km east of the Espinhaço Range. First, it was a new species of *Paepalanthus*, *P.oreodoxus* Andrino & Gonella, belonging to P.subgen.Xeractis (Körn.) Hensold, a lineage considered restricted to the ER and surrounding areas, but which “escaped” to these mountains further east (Andrino and Gonella 2021). More recently, a species known only from the holotype collected in the ER, *Paepalanthusminimus* Silveira, was rediscovered in these mountains and was combined into the genus *Giuliettia* due to its morphological characteristics (Andrino et al. 2024). These discoveries add to many other taxonomic novelties that have been described for the mountains of the medium Doce River basin in the last decade, many of them reflecting similar patterns of disjunction with the ER (Gonella et al. 2015, 2021; Siniscalchi et al. 2016; Mello-Silva 2018; Loeuille et al. 2019; Antar et al. 2021; Kollmann and Gonella 2021).

These mountains of the medium Doce River basin make up the João Pinto Geological Formation (Oliveira 2000), a set of outcrops of quartzitic rocks that form small mountains immersed in a matrix of seasonal forests of the Atlantic Forest domain, now severely transformed into pastures and plantations (Gonella et al. 2015). The quartzitic outcrops, particularly those above 800 meters, prominently feature campos rupestres, a vegetation that remains inadequately researched and protected in the region. Among these areas, Serra do Padre Ângelo deserves special attention, as most of the discoveries made in the region come from this complex of mountains south of the municipality of Conselheiro Pena. During the development of the project “Flora da Serra do Padre Ângelo”, an unidentified species of *Paepalanthus* was collected at one of the highest points of this mountain range, the Pico do Pinhão. After detailed studies, this species is described here as new and its affinities are discussed, along with data on its conservation status, reinforcing the urgency of taking action to protect the Serra do Padre Ângelo.

## ﻿Material and methods

The authors undertook fieldwork and collection efforts during an expedition in April 2022, during which the species was studied in its natural habitat and collected following traditional herborization techniques (Mori et al. 1985). Specimens were deposited at UB, MBML (paratype), and SPF (acronym according to Thiers 2024, continuously updated). Additional expeditions were conducted to locate the species on other isolated peaks within the type locality in October 2022 and February 2023, albeit without success. Subsequent investigations and consultations of materials deposited in herbaria (Reflora 2024; SpeciesLink 2024) yielded no previous collections of this species. The collected material underwent analysis using a stereomicroscope, considering both reproductive and vegetative characters according to specific terminology for describing Eriocaulaceae (Andrino et al. 2015; Andrino et al. 2023).

Conservation risk assessment was conducted following the criteria outlined by the International Union for Conservation of Nature (IUCN 2012). The distribution and occurrence map of the species and its related taxa were generated using QGIS software (QGIS Software Team 2024), employing layers provided by the Brazilian Institute of Geography and Statistics (IBGE 2024) and MapBiomas (2024). The coordinates of the new species, collected via GPS at the type locality, were used alongside the coordinates of related species obtained from data available in herbaria and virtual databases (JSTOR 2024; Reflora 2024; SpeciesLink 2024).

For the Scanning Electron Microscopy (SEM) analysis, seeds were retrieved from the selected holotype (*P.M. Gonella et al. 3402*). Seeds were immersed in water to facilitate the removal of fruit remnants, dried, and affixed onto stubs. They underwent gold coating using a Leica SCD 500 sputter coater. Subsequently, the specimens were examined and photographed using a scanning electron microscope JEOL-JSM-7001F.

## ﻿Results

### ﻿Taxonomic treatment

#### ﻿ Paepalanthus magnus

Taxon classificationPlantaePoalesEriocaulaceae

L.H.Rocha, Gonella & Andrino sp. nov.

38C5B89B-531E-5708-910F-30514BB88EE0

urn:lsid:ipni.org:names:77343388-1

**Figure 1.**
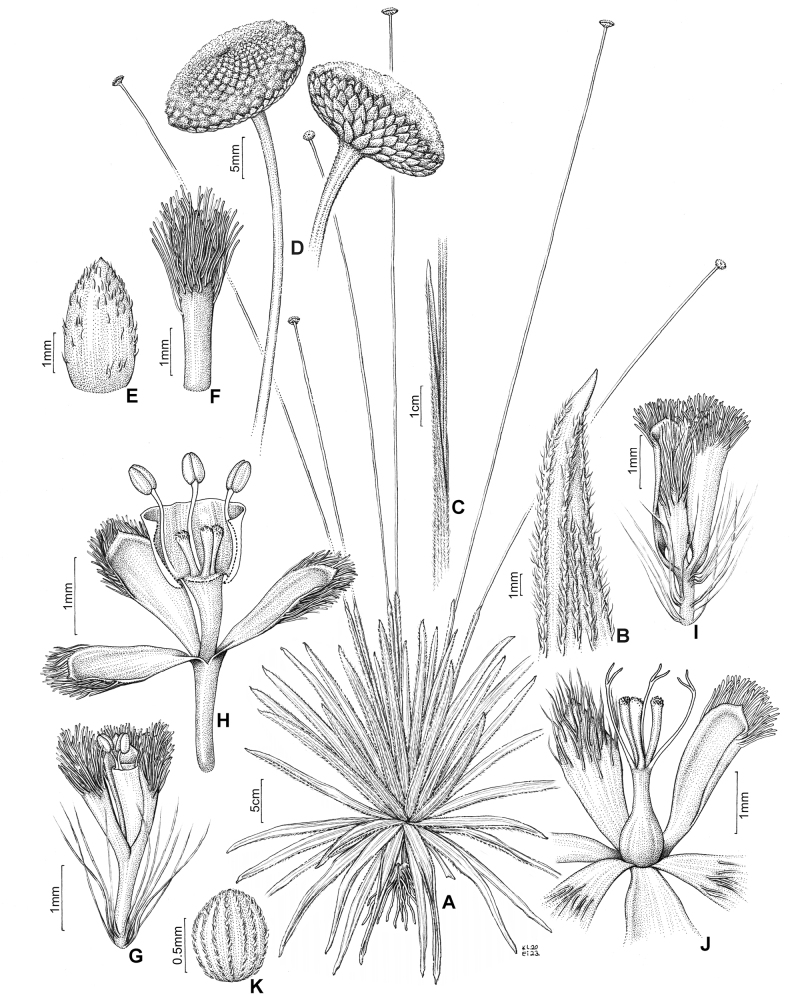
*Paepalanthusmagnus***A** habit **B** leaf apex **C** spathe, detail of the opening **D** capitula in dorsal (left) and ventral (right) view **E** involucral bract **F** floral bract **G** staminate flower in lateral view **H** staminate flower with sectioned corolla, exposing the stamens and pistillodes **I** pistillate flower in lateral view **J** pistillate flower with petals and sepals distended, exposing the gynoecium **K** seed with numerous appendices along the periclinal walls. Illustration by Klei Souza based on the holotype (*P.M. Gonella et al. 3402*).

Fig. 1 , 2 , 3 , 4 , 5

##### Type.

Brazil. Minas Gerais: Conselheiro Pena, Serra do Padre Ângelo, Serra do Pinhão, Pico do Pinhão, 19°15'21"S, 41°34'57.24"W, 1500 m elev., fl. & fr., 18 Apr 2022, *P.M. Gonella, L.H. Rocha, D.R. Couto, D.P. Cordeiro & E.C. Ribeiro 3402* (holotype: UB; isotype: SPF).

##### Diagnosis.

The new species is most similar to *Paepalanthusregelianus* Körn., with which it shares the pilose abaxial surface of the leaves. However, *P.regelianus* presents scapes that are about twice as long as the leaves and tomentose (*vs.* scapes at least 3 times longer than the leaves, and glabrous in *P.magnus*) and involucral bracts with a glabrous abaxial surface and ciliated margin (*vs.* pilose in *P.magnus*). Furthermore, the spathes of *P.regelianus* are shorter than its leaves, approximately half as long as the leaves (*vs.* spathes about as long as the leaves), present uniformly distributed trichomes (*vs.* along longitudinal nerves), and possess a short opening, no longer than 1 cm long (*vs.* opening distinctly longer, 2.5–6.0 cm long).

##### Description.

Terricolous or rupicolous perennial ***herb***, 55.0–94.5 cm high. *Roots* fibrous. ***Caudex*** present. ***Stem*** aerial, elongate, erect, thick, surrounded by marcescent leaves, 6.5–35.0 cm long. ***Leaves*** rosulate, 14.3–27.0 × 0.4–1.6 cm, linear-lanceolate, green, abaxial surface with trichomes ca. 4 mm long along the marked nerves, adaxial surface smooth and glabrous, margins ciliate, apex acute, mucronate. ***Inflorescences*** solitary per subtending leaf, axillary. ***Spathes*** 13.0–24.0 cm long, chartaceous, cylindrical, closed, abaxial surface pilose along the marked nerves (striate), adaxial (internal) surface glabrous, obliquely opened, opening 2.5–6.0 cm long, margin ciliate, apex acuminate. ***Scapes*** 50.0–89.0 cm long, 1–21 per plant (rosette), 5-costate, erect, glabrous, green to golden, free. ***Capitula*** 5.0–15.0 mm diam., white. ***Involucral bracts*** in 7–9 series, ca. 1.7–3.8 × 1.3–2.1 mm, ovate, castaneous, margin ciliate, abaxially pubescent, shorter than the flowers. ***Floral bracts*** ca. 3.5 mm long, linear-lanceolate, pigmented, densely pilose in the apical half with uniseriate trichomes ca. 2 mm long, margin ciliate. ***Flowers*** 3-merous, diclinous, arranged in concentric circles without clear organization. ***Staminate flowers*** ca. 3.5 mm long; pedicels ca. 1 mm long, pilose, with trichomes 2–2.5 mm long; sepals ca. 2.5–3 × 1 mm, oblanceolate, united in the base to up to 1/3 of length, castaneous with pigmentation concentrated in the center and becoming more translucent towards the margins, abaxial surface densely pilose in the apical 2/3, trichomes reducing in size towards the apex, adaxial surface glabrous, margin ciliate, apex acuminate; corolla tubular, ca. 2.5 mm long, free lobes ca. 0.3 mm long, entirely glabrous, hyaline; stamens epipetalous, filament ca. 1.5 mm long, anther dorsifixed, ca. 0.3 mm long; pistillodes papillose, ca. 1 mm long. ***Pistillate flowers*** ca. 4.5 mm long; pedicel ca. 0.5 mm long, densely pilose with long trichomes; sepals ca. 3.5 mm long, oblong, united only at the very base, castaneous with apex more strongly pigmented, adaxial surface glabrous, abaxial surface with apex slightly pilose, margin ciliate, apex acuminate-truncate; petals ca. 3 mm long, hyaline, base slightly pigmented, narrow obovate, free, adaxial surface pilose, abaxial surface glabrous, margin ciliate, apex acute; gynoecium with stigmatic and nectariferous branches emerging at the same height in the column, stigmatic branches ca. 1 mm long, apex bifid, nectariferous branches ca. 0.7 mm long, apex papillose, ovary ca. 1 mm diam, ovoid; *Seed* ca. 0.76 × 0.60 mm ovoid to ellipsoid, reddish, hilum acute, micropile obtuse, with numerous appendices with truncate apex along the periclinal walls.

##### Etymology.

The epithet “magnus” derives from the Latin “great”, “large”. This epithet was selected to denote the characteristic of the species being large in size, contrasting with the majority of Eriocaulaceae species found in the region where it occurs but also a reference to its larger size compared to its putative closest taxa.

##### Distribution and habitat.

**Figure 2.**
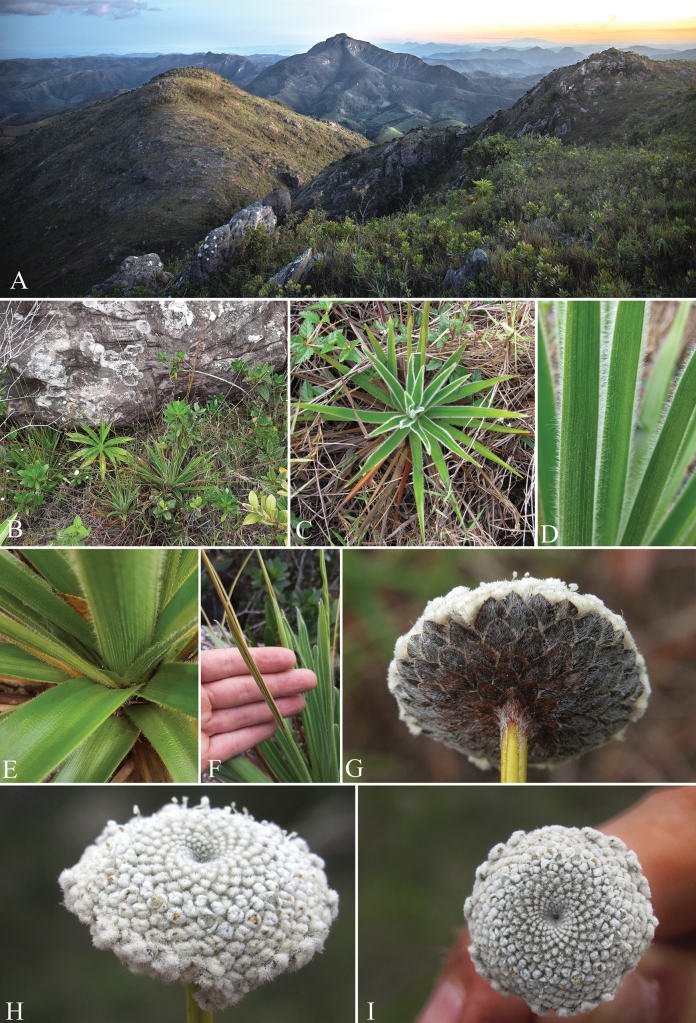
*Paepalanthusmagnus***A** habitat at Pico do Pinhão, with the Pico da Bela Adormecida (Pico do Padre Ângelo) in the background **B** habit among grasses and quartzitic rocks **C** rosette in detail **D** leaves, showing ciliate margin and striate abaxial surface **E** the base of the leaves, showing the adaxial surface and a scape enclosed by a spathe emerging from a leaf axil **F** spathe opening **G** capitulum in posterior view evidencing the involucral bracts **H** capitulum, lateral view **I** capitulum, frontal view. **A** by Lucian Medeiros **B–I** by PMG.

*Paepalanthusmagnus* is a microendemic species, found only at the top plateau of Pico do Pinhão (1540 m a.s.l.), one of the highest peaks of Serra do Padre Ângelo, a mountain complex located in the municipality of Conselheiro Pena, eastern Minas Gerais, southeastern Brazil (Fig. 4). The species was found at elevations above 1500 m a.s.l., forming a population of no more than 100 individuals growing directly on sandy soil among grasses or between large blocks of quartzitic rock, exposed to direct sunlight, in campos rupestres vegetation (Fig. 2A, B).

**Figure 3.**
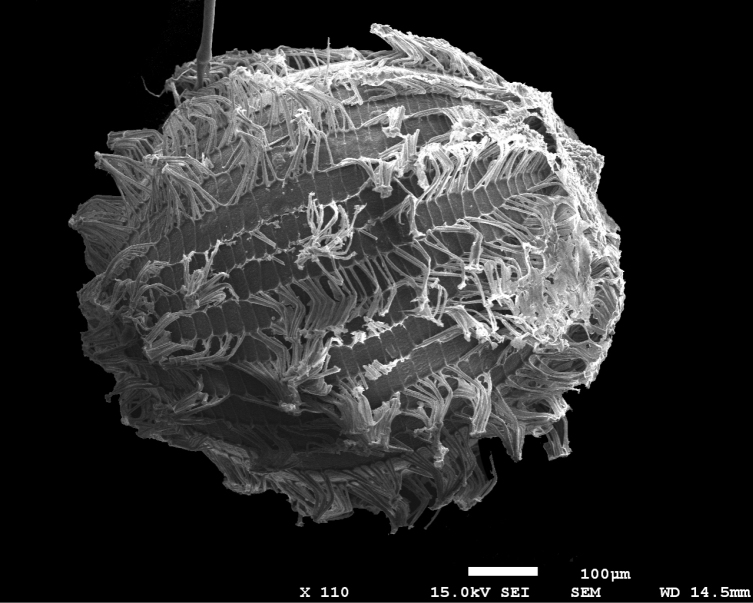
*Paepalanthusmagnus*. SEM micrograph of the seed coat (from the holotype, *P.M. Gonella et al. 3402*).

**Figure 4.**
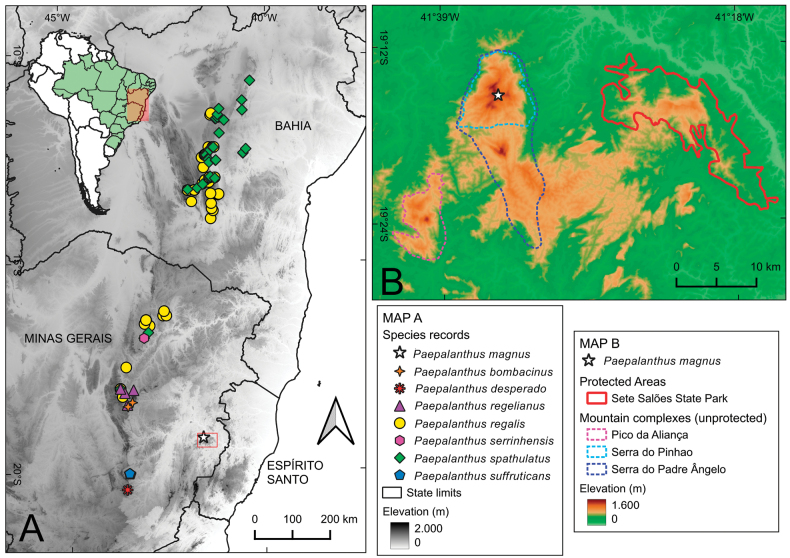
Distribution maps **A** distribution map of the new species and compared taxa cited in the text **B** distribution map of *P.magnus* at Serra do Padre Ângelo region, with other landmarks of the region indicated.

**Figure 5.**
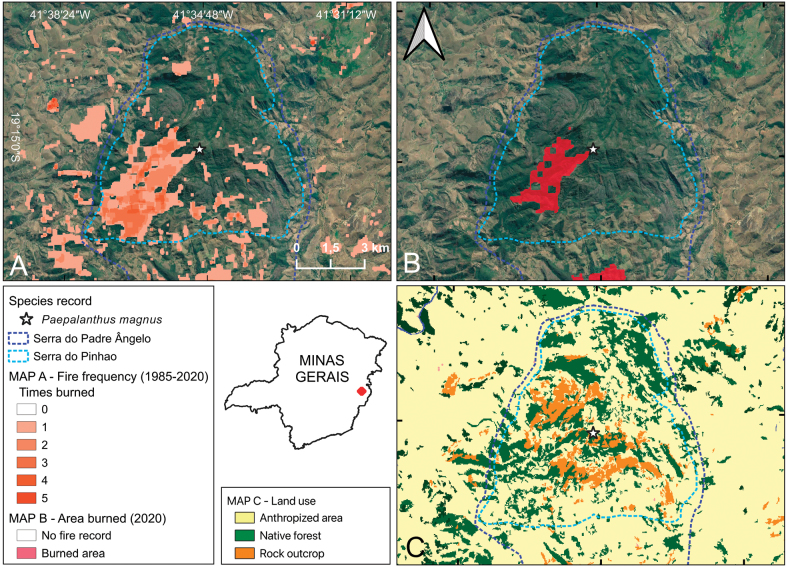
Conservation threats to *Paepalanthusmagnus***A** 35-year fire record (1985–2020) in the region of Serra do Pinhão, part of Serra do Padre Ângelo **B** fire record in the year 2020 **C** land use of the region. Data on fire and land use from MapBiomas (2024). **A**, **B** Map data ©2024 Google.

Pico do Pinhão is part of the northern massif of SPA, the Serra do Pinhão, whose culminating point is Pico do Sossego (1605 m), to the northwest of Pico do Pinhão. Expeditions to the former failed to find new populations of the species, which were also not found in the other higher peaks of the region, such as Pico da Bela Adormecida (also known as Padre Ângelo; 1550 m) and Pico da Aliança (1430 m), reinforcing the microendemic character of the species. At Pico do Pinhão, the campos rupestres are found at elevations above 1300 m. They are surrounded by a matrix of the Montane Seasonal Forest, which harbors the last individuals of the northernmost population of the endangered gymnosperm *Araucariaangustifolia* (Bertol.) Kuntze (Moura 1975), locally known as “pinhão” hence the name of the Serra. Such forest matrix, however, is severely degraded and is still subject to fires for land clearing and pasture formation (Fig. 5), as well as by the presence of cattle. The surrounding area is also severely invaded by alien species, especially the fern *Pteridiumaquilinum* (L.) Kuhn (Dennstaedtiaceae) and molasses grass *Melinisminutiflora* P. Beauv. (Poaceae).

##### Phenology.

Specimens were collected with flowers and fruits in April, which is by the end of the rainy season. The presence of old inflorescences with viable fruits, however, suggests that the flowering may occur since the beginning of the rainy season, which in the region starts in October/November.

##### Conservation status.

Preliminarily assessed as Critically Endangered – CR B1ab(iii) + B2ab(iii). The species is known from a single location in an area that is not protected, and which is subject to several ongoing threats to the quality of the habitat, such as deforestation, the presence of cattle, recurrent use of fires, and the presence of invasive species. Furthermore, species restricted to mountaintop habitats are especially vulnerable to the effects of climate change, especially intense droughts that may cause increased mortality (already observed in other taxa in the region) or intense rainfalls, which may cause landslides (already reported in the SPA following intense rainfall in the 2021/2022 rainy season). Arson fires are especially recurrent in the southwest of Serra do Pinhão (Fig. 5A) and are used by local farmers to renovate pastures and clear land for coffee and *Eucalyptus* plantations. These fires, however, often escape to native vegetation, causing the observed reduction of forest remnants and the intensification of invasion by the aforementioned alien species. The last of these intense fire events was in 2020 (Fig. 5B), also the year of another intense fire affecting the Pico da Bela Adormecida and its species (Andrino and Gonella 2021; Gonella et al. 2022; Andrino et al. 2024).

Since the species is known from a single location, it has an estimated AOO of 4 km^2^, and it does not have an associated EOO polygon, which, combined with the small population size and the listed ongoing threats, allow us to project a continuing decline in the quality of the habitat. Therefore, we suggest that the species should be declared Critically Endangered under the IUCN (2012) criteria. This preliminary assessment will be submitted to the Brazilian Flora authority of the IUCN Red List, coordinated by Centro Nacional de Conservação da Flora (CNCFlora), for validation.

##### Additional specimen examined (paratype).

Brazil. Minas Gerais: Conselheiro Pena, Serra do Padre Ângelo, complexo Serra do Pinhão, Pico do Pinhão, 18 Apr 2022, *D.R. Couto, P.M. Gonella, D.P. Cordeiro & L.H. Rocha-Pinto 6286* (MBML).

## ﻿Discussion

*Paepalanthusmagnus* unquestionably falls under the classification of *Paepalanthus* sensu Andrino et al. (2023). Its distinct seed coat morphology is characterized by rectangular or narrow hexagonal cells adorned with appendices exhibiting either a truncate apex or a “T” shape, devoid of micropapillae along the periclinal walls (Fig. 3). This unique feature set offers a robust foundation for the unequivocal placement of the species. Other distinctive characteristics include a habit featuring basal rosettes devoid of any reproductive or elongated axis (Fig. 1A, 2C), trimerous flowers with gamopetalous staminate flowers, and dialipetalous pistillate flowers. Additionally, its gynoecium exhibits free appendices, all releasing at the same level within the gynoecium (Fig. 1J). Its placement is also supported by the characteristic geographic distribution of the genus, centered in the campos rupestres of NE and SE Brazil (Andrino et al. 2023).

Based on the morphological features presented here, we suggest that *P.magnus* likely belongs to a lineage of species primarily distributed in the Chapada Diamantina, Bahia state (referred to as clade Q in Andrino et al. 2021). This lineage spans along the ER from the Chapada Diamantina to the northern region of the adjacent state of Minas Gerais (Fig. 4A), where some species are found in the biogeographic district of Grão Mogol and also on the Diamantina Plateau district (districts according to Colli-Silva et al. 2019). This lineage comprises species with a robust habit, often presenting a caudex, basal rosettes, and numerous long scapes emerging from leaf axils. The newly described species from Serra do Padre Ângelo likely aligns with this lineage based on morphological resemblance, although its placement needs to be properly tested.

Other species belonging to this lineage and likely closely related to *P.magnus* are *P.bombacinus* Silveira, *P.regelianus*, *P.regalis* Mart. ex Körn., *P.spathulatus* Körn., and *P.serrinhensis* Silveira. The comparison of *P.magnus* with these taxa, however, reveals unique morphological characteristics that make it a distinct addition to the diversity of the *Paepalanthus* genus in the region. The leaves and spathes with pilosity restricted to the veins (striate), the spathe as long as the leaves with long oblique opening, combined with glabrous green to golden scapes, and the capitula with bracts in more than seven series, are characteristics not shared by other species in this group, thus being important diagnostic features (Table 1). Furthermore, the observation of tufts of golden trichomes at the base of the leaves and on the sepals of staminate and pistillate flowers are characteristics that provide additional diagnostic information, a striking feature of the species that is also present in putative closely related species, such as *P.regelianus*.

**Table 1.** Diagnostic characters of the new species and the most similar taxa. Measurements of similar species were taken from herbarium specimens and literature (Andrino et al. 2015; Sano et al. 2010; Echternacht et al. 2021).

Character/Species	*P.magnus*	*P.bombacinus*	*P.regalis*	*P.regelianus*	*P.serrinhensis*	*P.spathulatus*
Stem	Elongate, 6.5–35.0 cm long	Restricted to the rosette	Restricted to the rosette	Restricted to the rosette	Restricted to the rosette to elongate, 12.0–17.0 cm long	Elongate, 4–10 cm
Leaf length	14.3–27.0 cm	14–24 cm	24.0–41.5 cm	15–26 cm	10.4–19.1 cm	9–32 cm
Leaf indumentum	Adaxial surface glabrous, abaxial surface pilose along conspicuous longitudinal nerves (striate)	Both surfaces glabrescent to pubescent	Glabrous on both surfaces	Adaxial surface glabrous to glabrescent, abaxial surface uniformly pilose	Glabrous to glabrescent on both surfaces	Glabrous
Spathe length	13–18 cm	11–15 cm	7.4–30.0 cm	8–13 cm	5.5–6.7 cm	4.5–6.5 cm
Spathe indumentum	Pilose along longitudinal nerves (striate)	Pilose along longitudinal nerves (striate)	Glabrous	Uniformly pilose	Glabrous	Glabrous
Spathe opening	25–60 mm long	15 mm long	40 mm long	10 mm long	15 mm long	4.5–8.0 mm long
Scape length	50–89 cm	45–50 cm	18–62 cm	36–45 cm	17–32 cm	18–29 cm
Scape indumentum	Glabrous	Tomentose	Glabrous	Tomentose	Glabrous to sparsely pilose	Glabrous
Involucral bracts number of series and shape	7–9 series, ovate, shorter than the flowers	4–6 series, narrowly ovate to deltoid, shorter than the flowers	7 series, deltoid, shorter than the flowers	4–5 series, triangular, shorter than the flowers	Up to 4 series, ovate, shorter than the flowers	3–4 series, lanceolate, surpassing the height of the flowers
Involucral bracts abaxial indumentum	Pubescent	Lanuginose	Glabrous	Pubescent	Pubescent to glabrescent	Glabrous

Besides the aforementioned unique features of *P.magnus* in comparison with the putative closely related species, these taxa can be distinguished by their unique features: *P.bombacinus* can be recognized by its involucral bracts that are densely tomentose (lanuginose) abaxially; *P.regelianus* (including the synonym *P.coronarius* Silveira; Andrino et al. 2022) is distinctive in having densely pilose scapes; *P.regalis* is not to be confused by its glabrous leaves, spathes, and scapes, as well as by its unique laterally flattened capitula; *P.serrinhensis* presents a slender habit, glabrous leaves and small capitula with ovate involucral bracts; and *P.spathulatus* presents spatulate glabrous leaves and distinctive lanceolate bracts with an acute apex that surpass the length of the flowers (see Table 1 for further distinctive characters).

Among the specimens of *P.regelianus* studied for this work, there was a specimen (*Irwin 27519*) with many duplicates spread in different herbaria with different identifications, including *P.regalis* (at K), *P.bombacinus* (F), and *P.desperado* Ruhland (NY, US). *Paepalanthusdesperado* is a remarkable species known only from the type and a few recent collections, all from around Lavras Novas (district of Ouro Preto, in the Iron Quadrangle; Fig. 4A). This species bears similar spathes with the new species, which are as long as the leaves, present trichomes disposed on longitudinal ridges, and a large oblique opening. *Paepalanthusdesperado*, however, is a slenderer species, with a more delicate elongate stem, narrower and more delicate leaves, and less robust capitula with fewer series of involucral bracts. Such species appears to be closely related to another more delicate species, *P.suffruticans* Ruhland, microendemic to the Serra do Caraça, also in the Iron Quadrangle (Fig. 4A). Based on general morphology, we hypothesize that these species might not be closely related to the new species or the group to which it belongs, but molecular phylogenetic studies could better test this hypothesis.

The discovery of *Paepalanthusmagnus* in the Serra do Padre Ângelo reinforces a pattern observed in other mountainous regions, such as the Espinhaço Range. In these high-altitude environments, many species of Eriocaulaceae are microendemic, often exclusive to a single mountaintop (Costa et al. 2023). This distribution pattern can be explained by the fragmentation of landscapes, which isolate lineages in areas of high topography with dystrophic soils, fostering speciation (Vasconcelos et al. 2020). Analyzing the pattern of microendemism on mountaintops in the Serra do Padre Ângelo may provide additional insights into the evolution of plants in the region, as this pattern is repeated in other campo rupestre lineages from different angiosperm families, such as Asteraceae, Begoniaceae, Bromeliaceae, and Droseraceae, among others.

While species closely related to *P.magnus* are restricted to the northern ER (Fig. 4A; Andrino et al. 2021), other Eriocaulaceae species from SPA have a closer connection with the southern ER, such as the Iron Quadrangle (Andrino and Gonella 2021) or the Diamantina Plateau (Andrino et al. 2024). This pattern of geographic disjunction is intriguing and suggests the possibility of a myriad of historical events and environmental factors influencing the distribution of these species. This discovery contributes a piece to the intricate puzzle of the flora in the easternmost campos rupestres of Minas Gerais, where a diverse assembly of species with distinct biogeographic histories converge. While this finding enriches our understanding, it’s important to note that it is just one part of the larger puzzle. Future research, such as genetic and biogeographic studies, may properly test this hypothesis and provide a deeper understanding of the phylogenetic relationships within clade Q of *Paepalanthus* and the evolution of Eriocaulaceae in the northern Espinhaço and disjunct campos rupestres areas, such as the João Pinto Formation.

The discovery of yet another new and potentially threatened species in the quartzitic mountains of the João Pinto Formation reinforces the recognition of this area as an endemism center of the campos rupestres flora, as more than 30 new species have been described in the last decade (see Leme et al. 2023 and references therein). However, the situation of these species must be viewed with concern, particularly regarding their conservation. These mountains are only protected by a single State Park (Parque Estadual de Sete Salões), which does not encompass any of the highest peaks of the region (Fig. 4B). Instead, these peaks are situated in unprotected public lands surrounded by small farmlands, and criminal and uncontrolled arson fires continue to threaten native species and exacerbate the invasion by alien species. Urgent action is needed to establish a conservation strategy for these higher areas, with a proposition for the creation of a protected area in the Serra do Padre Ângelo currently under discussion through the Territorial Action Plan “Capixaba-Gerais” (“PAT Capixaba-Gerais”). The success of any conservation effort, however, relies on the active involvement of local and regional administrations and the engagement of local communities to ensure the long-term preservation of this region and its unique biodiversity.

## Supplementary Material

XML Treatment for Paepalanthus magnus

## ﻿Additional information

### Author ORCIDs

Luiz Henrique Rocha https://orcid.org/0009-0004-9444-8849

Paulo Minatel Gonella https://orcid.org/0000-0001-8332-5326

Caroline Oliveira Andrino https://orcid.org/0000-0003-1107-5692

### Data availability

All of the data that support the findings of this study are available in the main text.
